# Scientific telephone: The cautionary tale of the global coverage of lichens

**DOI:** 10.1093/biosci/biae048

**Published:** 2024-07-04

**Authors:** Katherine H I Drotos, Douglas W Larson, R Troy McMullin

**Affiliations:** Department of Integrative Biology at the University of Guelph, Guelph, Ontario, Canada; Department of Integrative Biology at the University of Guelph, Guelph, Ontario, Canada; Canadian Museum of Nature, Research and Collections, Ottawa, Ontario, Canada

**Keywords:** science communication, citation practices, global vegetation, terrestrial coverage

## Abstract

Scientific history has many examples of profound statements that are later found to be unsubstantiated. The consequences of such misinformation can be dire. In the present article, we present a case where an unevidenced estimate of global lichen coverage proliferated through both scientific literature and popular media. We traced this estimate to a non-peer-reviewed publication from 1987. We found 76 academic articles (collectively cited 4125 times) and 13 other academic documents citing the statistic, citation chains without source attribution, and instances where the number or context was changed. We also found the statistic 37 times in popular media, which is especially concerning, given that these media communicate science to the broader public. We demonstrate how an unevidenced statement can spread, change through time, and ultimately be repeated without demand for evidence. We hope this case unplugs the telephone and provides a cautionary tale for researchers to ensure critical evaluation of citation and communication practices.

The history of science is filled with examples of attractive ideas that are later shown to be false, because of either mistake or malice. Early recordings of ancient philosophers often contain generalizations about the natural world based on what they could see and measure at the time. In the current era, some of these unsupported ideas originate from unsubstantiated assumptions or poor research. For example, the idea that babies do not feel pain came from assumptions about human baby development and poorly conducted experiments (Rodkey and Pillai Riddell 2013, Segner 2016); the idea that lemmings jump off cliffs en masse was orchestrated by a documentary crew (Chitty 1996, Woodford 2003); and the idea that vaccines cause autism came primarily from a single study that was later retracted because of both ethical and study design failures (Eggertson 2010, Rao and Andrade 2011).

Given what the field of science is and purports to be, instances such as these remind us that the evaluation of evidence and the interpretation thereafter is critical. Although the consequences of these false claims may vary in their ultimate severity, no field is immune. Rare also, as was noted by Chitty (1996), is the ability to follow such a claim from its inception to its impact. In the present article, we do exactly this by presenting a case study concerning a statement about the global coverage of lichens. The statement in question differs from the examples above in that there was no initial evidence (not even poor evidence) to supposedly support the claim. Instead, this claim sprang into being by conjecture without substantiation and then spread despite a lack of evidence. The path of this statement follows some of the examples above, however, in that it was allowed to freely disperse largely unchecked for a period of time. We present the story of this statement, quantify its spread, and discuss some potential consequences of unsubstantiated claims of this nature.

## The 8% story

**Figure 1.**
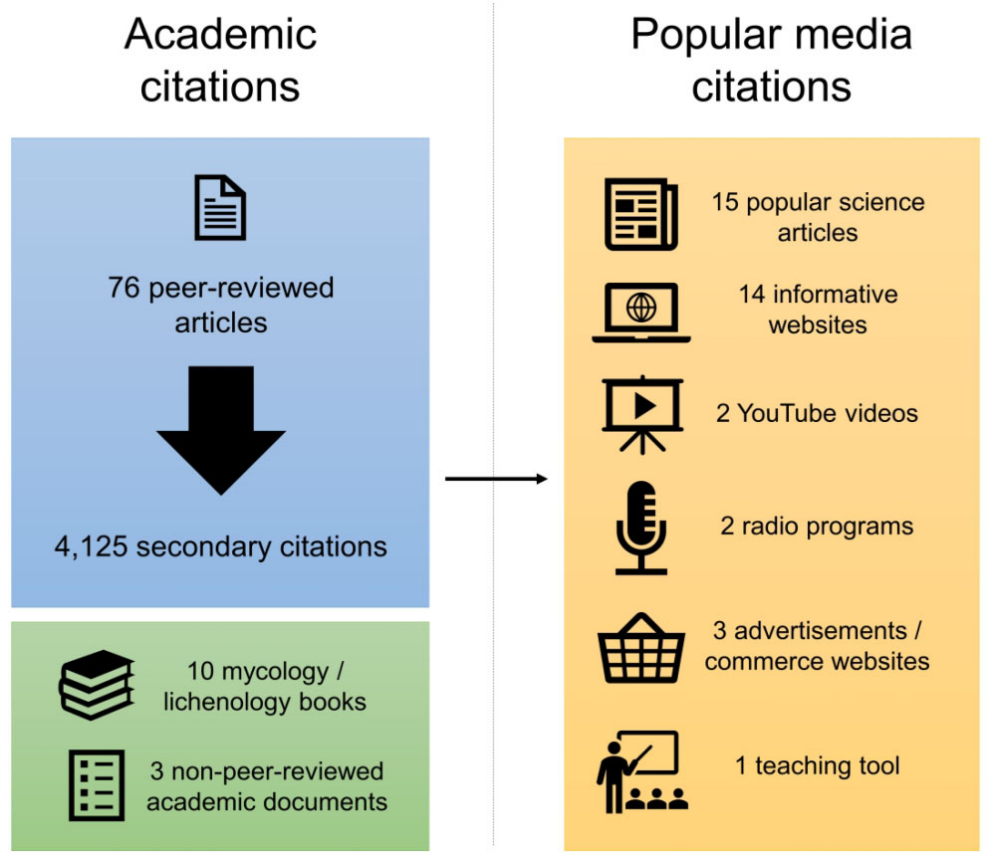
Citations are grouped as appearing in academic or popular media. Popular media references are delineated by format. The horizontal arrow represents the flow of information from published literature to popular media.

It is currently and commonly claimed that 8% of the world is covered in lichens. Eighty-nine academic and 37 popular media items contain some variation of this statement (figure 1), with at least 5600 secondary citations for the former group (see supplementary materials). With any such claim, we can immediately ask, *How do we know this?* This question has a simple answer: We don't. If we were to begin with the question *How much of the world is covered in lichens?* an evidence-based answer would involve a massive global effort including satellite data, ground surveys, modeling, and international coordination. In short, such work has not yet been done at a global scale. So how did the 8% claim come to be, and why is it repeated so often?

In 1974, when one of the authors (DWL) was a graduate student, Vernon Ahmadjian—then a prominent figure in global lichenology—gave a seminar to a group of graduate students at McMaster University. At one point, he stated that lichens were understudied globally, despite 8% of the terrestrial surface of the Earth being dominated by lichen. The comment was presumably made to focus attention rather than to attract peer review. No one challenged the number, because it was plausible—unlike, say, 0.8% or 80%. None of the students asked where the number came from. The number did not go into print of any kind at this point.

In 1986, DWL attended a conference in Germany organized to summarize progress and problems in lichenology at the time. He gave a presentation entitled “The absorption and release of water by lichens.” He repeated the 8% statistic to emphasize—as Ahmadjian had done—that lichens were overlooked by biologists. DWL, however, made the mistake of not attributing the number to Ahmadjian during the talk. The number drew no attention from those in attendance.

In 1987, the conference organizers decided to publish a non-peer-reviewed summary of the conference by having the oral presentations presented as brief articles. DWL included the 8% statistic in the introduction to the summary (table 1; Larson 1987), but again failed to include a personal communication citation to Ahmadjian, and the publisher of the conference proceedings did not ask for a citation for the number. Again the 8% went largely unnoticed, and the career of DWL in lichenology ended as his work shifted to other areas of study.

**Table 1.** Quotes from the original two papers that are frequently cited as references for 8% of the world's terrestrial surface being dominated by lichen.

Author	Year	Exact quote	Total number of academic citations for the 8% statistic
Larson	1987	“When one considers the roughly 8% of the Earth's terrestrial surface that is lichen dominated, one is impressed with how well this particular alga–fungus mutualism has extended the range of its previously free-living drought-sensitive ancestors.”	38
Ahmadjian	1995	“According to Douglas W. Larson of the University of Guelph in Ontario, Canada, approximately 8% of the Earth's terrestrial surface has lichens as its most dominant lifeforms.”	33

*Note:* The number of citations refers to citations for the 8% statistic only. For both papers, most of their citations were for this purpose.

But the career and status of Ahmadjian as a global leader in lichenology grew, and in an invited letter in *BioScience*, he wrote about the global importance of lichens (Ahmadjian 1995). In it, he claimed that lichens were the dominant organism in 8% of the terrestrial habitats on Earth and attributed the number to Larson, though he did not include a date in the reference (table 1). Because of Larson's omission of a personal communication citation in his own work, Ahmadjian evidently did not realize that the 8% statement originated with his own much earlier lecture. That invited letter was the first time a specific reference was provided for the statistic (minus a date) even though the 1987 paper itself provided no evidence and was not peer reviewed. But the reputation and status of Ahmadjian made the claim seem reasonable—even plausible. As a result, interested parties started to repeat the claim, sometimes attributed to Ahmadjian and sometimes to Larson. Because so many inclusions of the 8% statement cite Ahmadjian and because no date was assigned to his citation of Larson (and, therefore, some authors may have assumed it was a personal communication), we will discuss both pieces as origin points for the statement.

**Figure 2.**
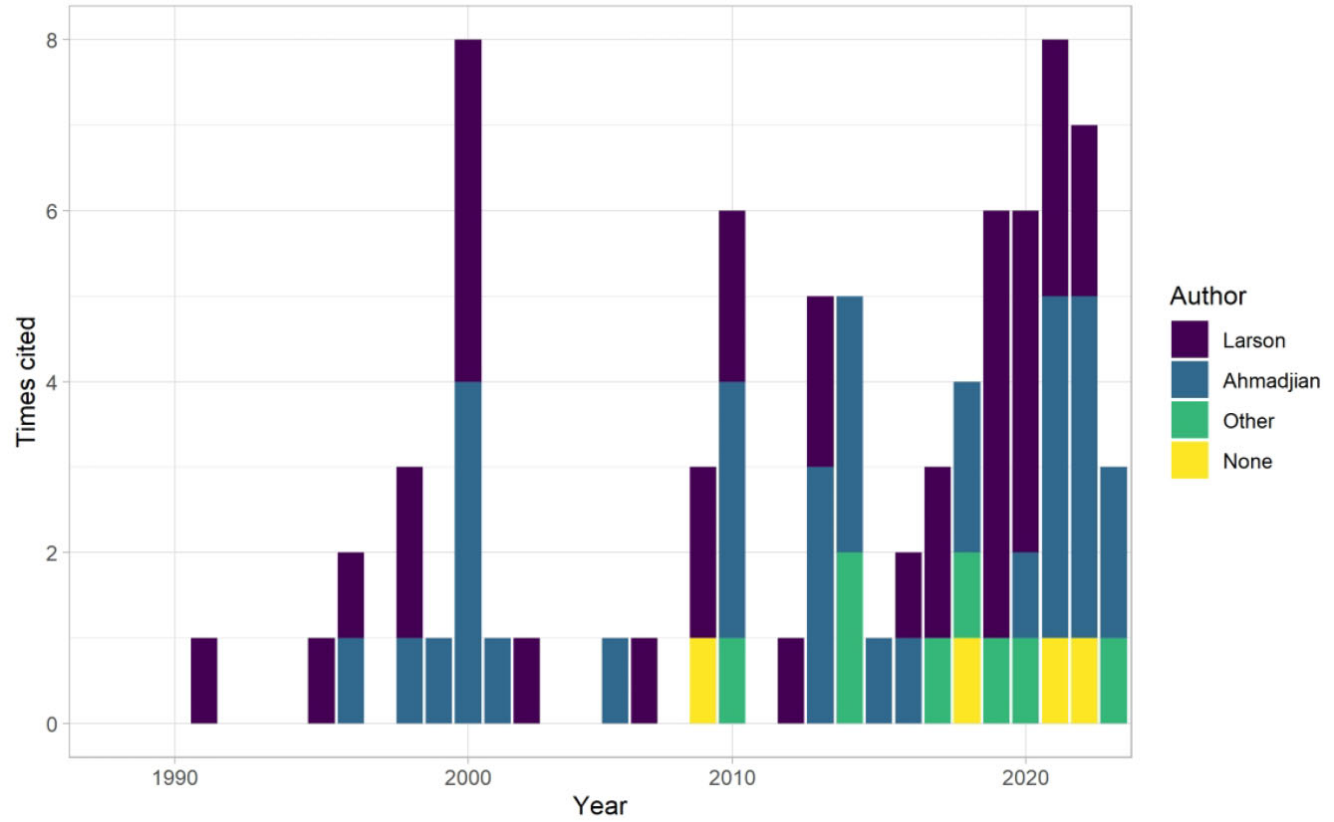
Citations for some variation of the 8% statistic in peer-reviewed articles over time. In instances where a publication included multiple sources, all were counted individually (i.e., the total count is 80 because four publications included two sources). Citations by year were tracked through the Web of Science.

Nearly 30 years later, the claim—now worded in different ways—has been repeated throughout the scientific literature, in popular print, and in many forms of electronic media without anyone asking for either definitions of the word *dominant* or for evidence to support the claim. The academic citations for the 8% statement include 76 papers that have, in turn, been collectively cited at least 4125 times (a few for the 8%, but mainly for other purposes; table 2). There are nine instances where neither Larson nor Ahmadjian are cited directly: Another author is cited, or no citation is given. With the former case, there is sometimes a chain of citations (two to four deep) that eventually leads to one of these original papers. Citations in peer-reviewed articles have generally increased through time (figure 2). In addition, the statistic is included in at least 13 books and other non-peer-reviewed academic documents, 3 of which are popular lichenology texts (Ahmadjian 1993, Brodo et al. 2001, Beckett et al. 2008, Seaward 2008). These texts have been cited collectively many hundreds of times (per Web of Science and Google Scholar), and it is possible that some of those citations are for the 8% statistic. It is more challenging to track citations from books in this manner, but it is worth noting that they are widely used by both experts and students of lichenology.

**Table 2.** Total instances found at the time of this publication for the inclusion of the 8% statistic. See the supplementary materials for full literature list.

	Peer-reviewed articles		Books and other academic documents		
Source	Number of articles	Secondary citations	Number of items	Secondary citations^c^	Number of popular media items
Larson 1987	35	1691	3^b^	1247	0
Ahmadjian 1995	32^a^	954	1	23	1
Other sources	5	1420	0	n/a	2
No citation	4	60	9	258	34
Total	76	4125	13	1528	37

*Note:* Author denotes who was cited for the statistic. The number of items is the raw count; secondary citations refer to how many times those publications have been collectively cited. In some cases, the number of secondary citations was undetermined because of being unlisted in Web of Science. In instances where more than one citation was included, only the older citation was counted (e.g., Larson 1987 over any additional citation).

a There was a single citation for Ahmadjian 1993, which is included.

b This includes Ahmadjian 1995 citing Larson.

c Some of these are vast underestimates, because accurate citations for Brodo et al. 2001, Nash 2008, and Tzovaras 2018 were unavailable.

Many authors use the 8% statistic with its original intent; however, there are several instances of considerable change. Of the 76 papers, at least 12 did not state it as an estimate but, rather, with certainty. Seventeen papers changed the number itself (anywhere from 6% to 10%, and sometimes with a range), even when citing one of the original sources. At least 18 papers changed the original statement from *ecosystem dominance* to another term—frequently *abundance, cover*, and *diversity*. Its inclusion is primarily in the introduction of these articles as a “factoid.” This suggests that the number is viewed as a standard piece of information to inform the reader about lichen biology.

The term *dominated* in the original document was not explicitly defined. Dominance is often context specific, however, and could have multiple interpretations. For example, dominance can refer to raw abundance, relative abundance, cover, impact on the community, and more (Avolio et al. 2019). It is probable that, in most cases, the change from *dominance* to another term is an honest mistake. A key takeaway is to be clear on the intent behind such words, where context of the science and the reader's background influence the interpretation.

We found 37 inclusions of the statistic in popular or other nonacademic media (figure 1). Only three included a citation, and only one of these was to one of the original sources. Most of the inclusions were for either popular science articles discussing a recent lichenology article (15) or for informative websites on lichen biology (14). These numbers may seem low, but lichens are infrequently discussed in public media. Including this statistic as a fundamental truth in any popular media relating to lichens is problematic, because the odds of nonscientists (or nonlichenologists, for that matter) interacting with any media about lichens is very low. It would stand to reason that any piece of media discussing lichen biology may be foundational or at least critical for the audience.

### Unplugging the telephone: Mistakes made and methods to avoid them

How does a statement become regarded as “true” through repetition? For one, it must be believable. Eight percent of terrestrial vegetation being lichen dominated is plausible, whereas 0.8% or 80% are much less so. It also must serve some purpose; given how far the statistic has spread and how often it is repeated, there is a desire for such a number. Regardless of the biological implications, it is clearly important to those who care about lichens, or it would not so readily be repeated. Finally, it is also likely a statement that is difficult to falsify. Discussion of any biological entity at a global scale includes some amount of speculation; the key is whether this speculation is based on evidence.

There are two important points to this story. One is that Larson (and to some extent, Ahmadjian) failed to ask, clarify, or properly cite where the 8% statement came from or what evidence it was based on, and so the number entered the literature without basis. The second point is that many authors since then have included the number without asking what evidence supports it and, in some cases, have not cited an original source or have changed the statement itself. Both errors have led to 8% becoming the default answer to the question of how much of the world is covered in lichens or similar.

The path and pervasiveness of the 8% statistic is reminiscent of the game telephone, where a message is passed through multiple people and typically becomes increasingly misconstrued the more it is repeated. The statistic's repeated use led it to become enshrined as “fact,” with little regard for where it originated. The papers including the statistic are obviously widely read among lichenologists and beyond (given their collective secondary citations of 4125), and this correlates with the citation chains that have gone unchecked. It is difficult to say how much research has relied on this number or included it as an assumption. It is even more difficult to quantify the consequences of its use. Perhaps most obviously, it implies that an assessment of global lichen coverage has already been conducted when it has not.

Lichens are critical components of terrestrial ecosystems. In addition to their well-known role as a critical winter food source for many caribou populations (Thomas and Hervieux 1986, Ferguson et al. 2001, Thompson et al. 2015), lichens affect albedo (Belnap 1995, Stoy et al. 2012, Rutherford et al. 2017) and erosion (Belnap and Büdel 2016) of the landscape. They provide or contribute habitat for both invertebrates (Stubbs 1989, Behan-Pelletier et al. 2008, Bates et al. 2012, Muñoz-Li and Capote 2021) and vertebrates (Bent 1940, Hayward and Rosentreter 1994, Graves and Forno 2018). They are also used as indirect indicators of ecological metrics of interest, such as succession (Whittet and Ellis 2013, Miller et al. 2020) and pollution (Van Dobben et al. 2001, Malaspina et al. 2018, Kousehlar and Widom 2020). In addition, recent work on the deeper nature of the symbiosis and evolutionary history of lichens has propelled them into the spotlight (Spribille et al. 2016, Spribille 2018, Allen and Lendemer 2022, Sanders 2023, Scharnagl et al. 2023) and argued that lichens may serve as model systems for interesting ecological and evolutionary investigations. It is critical then that this new era of lichenology is built on evidence-based foundations on which further work relies.

Lichenology is hardly the only subject to experience widespread claims that are later realized to be unsupported. Many cases, such as those related to human health, have serious consequences. Avoidance of these unsupported claims begins with the publication of the original research, where it is important to be clear about speculation versus evidence, to not overstate the significance of the findings, and to place them in the context in which they apply. At the other end of the publication pipeline, investigators should always strive to find the original or ultimate source of information and validate it before citing it. None of this is to say that such estimates should be abandoned entirely. Rather, it means that care should be taken when citing claims that are not supported by empirical data and that transparency and context are key.

Science communication, between any and all stakeholders, should be based on evidence. Missing citations or miscitations are problematic because it makes it difficult for anyone—researchers and nonexperts alike—to validate claims. It also risks the erosion of public trust in science. Some segments of the general population are already wary of science and scientists, and instances such as these can further that distrust (Fiske and Dupree 2014, Funk and Rainie 2015). It is also arguably our responsibility as scientists to ensure that the information we relay to journalists and to the broader public is accurate.

With this case study, we hope to unplug the telephone of a repeatedly cited and unsubstantiated statement, and that it may serve as a reminder for critical evaluation of citation and communication practices in science. Information spreads through many mechanisms, and so as scientists we must take care with what we communicate and how. We as lichenologists choose to see this particular instance as an opportunity: We actually don't know how much of the world is covered in lichens, and this is an exciting mystery to solve.

## Supplementary Material

biae048_Supplemental_File

## Author Biography

Katherine H. I. Drotos (kdrotos@uoguelph.ca) and Douglas W. Larson are affiliated with the Department of Integrative Biology at the University of Guelph, in Guelph, Ontario, Canada. R. Troy McMullin is affiliated with the Canadian Museum of Nature, Research and Collections, in Ottawa, Ontario, Canada.
