# Supplementary material for: Scientific telephone: The cautionary tale of the global coverage of lichens
Source: Bioscience. 2024 Jul 4;74(7):473–7. doi: 10.1093/biosci/biae048 (PMC11328143; doi:10.1093/biosci/biae048)
Supplement: biae048_Supplemental_File [file biae048_supplemental_file.pdf]

Peer-reviewed works

Date publi	Citation	Percent coverag	Article type	Publication	Authors	Title	Times Cite	DOI or link
1991	Larson 1987	8%	Peer-reviewed article	Annual Review of Plant Physiol	Honegger, R	FUNCTIONAL-ASPECTS OF THE LICHEN SYMBIOSIS	246	10.1146/annurev.pp.42.060191.003005
1995	Larson 1987	8%	Peer-reviewed article	Canadian Journal of Botany	Honegger, R	Experimental studies with foliose macrolichens: fungal responses	22	https://www.webofscience.com/wos/woscc/full-record/WOS:A1995TC46200080
1996	Ahmadjian 1995	8%	Peer-reviewed article	Current Science Association	Hans Raj Negi and Madhav Gadgil	Patterns of distribution of macrolichens in western parts of Nand	10	https://www.webofscience.com/wos/woscc/full-record/WOS:A1996VM57200018
1996	Larson 1987	8%	Peer-reviewed article	Annals of Botany	VALLADARES, FERNANDO ; SANCHO, LEOPOLDO G ;	Functional analysis of the intrathalline and intracellular chlorophy	25	10.1006/anbo.1996.0144
1998	Larson 1987	8%	Peer-reviewed article	The Lichenologist	Honegger, R	The lichen symbiosis - what is so spectacular about it?	95	https://www.webofscience.com/wos/woscc/full-record/WOS:000074346700001
1998	Larson 1987 and Ahmadjian 1995	8%	Peer-reviewed article	Arctic and Alpine Research	Lange, OL; Hahn, SC; Meyer, S; Tenhunen, JD	Upland tundra in the foothills of the Brooks Range, Alaska, USA: L	24	10.2307/1551972
1999	Ahmadjian 1995	8%	Peer-reviewed article	J Plant Physiol Vol	Lange, OL; Green, TGA; Reichenberger, H	The response of lichen photosynthesis to external CO2 concentra	36	10.1016/S0176-1617(99)80204-1
2000	Ahmadjian 1995	8%	Peer-reviewed article	Journal of Biosciences	Negi, HR	On the patterns of abundance and diversity of macrolichens of Ch	18	10.1007/BF02703790
2000	Ahmadjian 1995	8%	Peer-reviewed article	Journal of Geophysical Researc	Kuhn, U; Kesselmeier, J	Environmental variables controlling the uptake of carbonyl sulfide	27	10.1029/2000JD900436
2000	Ahmadjian 1995	8%	Peer-reviewed article	Atmospheric Environment	Kuhn, U; Wolf, A; Gries, C; Nash, TH; Kesselmeier, J	Field measurements on the exchange of carbonyl sulfide betweer	22	10.1016/S1352-2310(00)00235-1
2000	Ahmadjian 1995	8%	Peer-reviewed article	Current Science	Hans Raj Negi and D. K. Upreti	Species diversity and relative abundance of lichens in Rumbak cat	19	https://www.webofscience.com/wos/woscc/full-record/WOS:000087028400019
2000	Larson 1987	8%	Peer-reviewed article	New Phytologist	Palmqvist, K	Carbon economy in lichens	205	10.1046/j.1469-8137.2000.00732.x
2000	Larson 1987	8%	Peer-reviewed article	Catena	Chen, Jie ; Blume, Hans-Peter ; Beyer, Lothar	Weathering of rocks induced by lichen colonization - a review	363	10.1016/S0341-8162(99)00085-5
2000	Larson 1987	8%	Peer-reviewed article	Plant, Cell and Environment	Palmqvist, K ; Sundberg, B	Light use efficiency of dry matter gain in five macro-lichens: relati	107	10.1046/j.1365-3040.2000.00529.x
2000	Larson 1987	8%	Peer-reviewed article	Rangifer	Crittenden, PD	Aspects of the ecology of mat-forming lichens	66	10.7557/2.20.2-3.1508
2001	Ahmadjian 1995	8%	Peer-reviewed article	Journal of Experimental Botany	Lange, OL; Green, TGA; Heber, U	Hydration-dependent photosynthetic production of lichens: what	104	10.1093/jexbot/52.363.2033
2002	Larson 1987	8%	Peer-reviewed article	Lichenologist	Storeheier, Pål Vegar ; Mathiesen, Svein D ; Tyler, Ni	Nutritive value of terricolous lichens for reindeer in winter	74	10.1006/lich.2002.0394
2005	Ahmadjian 1995	8%	Peer-reviewed article	Oecologia	Lange, OL; Green, TGA	Lichens show that fungi can acclimate their respiration to season	74	10.1007/s00442-004-1697-x
2007	Larson 1987	8%	Peer-reviewed article	Oecologia	Sari Stark ; Minna-Maarit Kytöviita ; Antje B. Neuma	The phenolic compounds in Cladonia lichens are not antimicrobia	34	10.1007/s00442-006-0644-4
2009	Larson 1987	8%	Peer-reviewed article	Biologia Plantarum	Backor, M ; Loppi, S	Interactions of lichens with heavy metals	154	10.1007/s10535-009-0042-y
2009	Larson 1987	8%	Peer-reviewed article	Functional Ecology	Minna-Maarit Kytöviita ; Sari Stark	No allelopathic effect of the dominant forest-floor lichen Cladonia	24	10.1111/j.1365-2435.2008.01508.x
2009	no citation	8%	Quick Guide	Current Biology	François Lutzoni and Jolanta Miadlikowska	Lichens	48	10.1016/j.cub.2009.04.034
2010	Haas & Purvis 2006	6%	Peer-reviewed article	Microbiology	Gadd, G.M.	Metals, minerals and microbes: geomicrobiology and bioremedia	1145	10.1099/mic.0.037143-0
2010	Ahmadjian 1993	8%	Peer-reviewed article	Geomicrobiology Journal	Kidron, Giora J ; Temina, Marina	Lichen Colonization on Cobbles in the Negev Desert Following 15	9	10.1080/01490450903490805
2010	Ahmadjian 1995	8%	Peer-reviewed article	Symbiosis	Muggia, L; Zellnig, G; Rabensteiner, J; Grube, M	Morphological and phylogenetic study of algal partners associate	37	10.1007/s13199-010-0060-8
2010	Ahmadjian 1995	8%	Peer-reviewed article	Polar Biology	Selbmann, L; Zucconi, L; Ruisi, S; Grube, M; Cardinal	Culturable bacteria associated with Antarctic lichens: affiliation ar	71	10.1007/s00300-009-0686-2
2010	Larson 1987	8%	Peer-reviewed article	Soil Biology and Biochemistry	Stark, Sari ; Männistö, Minna K ; Smolander, Aino	Multiple effects of reindeer grazing on the soil processes in nutrie	20	10.1016/j.soilbio.2010.08.001
2010	Larson 1987	8%	Peer-reviewed article	Journal of Chemical Ecology	NYBAKKEN, Line ; HELMERSEN, Anne-Marit ; GAUSL	Lichen Compounds Restrain Lichen Feeding by Bank Voles (Myod	32	10.1007/s10886-010-9761-y
2012	Larson 1987	8%	Peer-reviewed article	Landes Bioscience	Rodriguez, CM; Bennett, JP; Johnson, CJ	Lichens: Unexpected anti-prion agents?	7	10.4161/pri.6.1.17414
2013	Ahmadjian 1995	8%	Peer-reviewed article	Phytochem Rev	Shrestha, G; St. Clair, LL	Lichens: a promising source of antibiotic and anticancer drugs	141	10.1007/s11101-013-9283-7
2013	Ahmadjian 1995	8%	Peer-reviewed article	Environmental Science: Proces	Mestrot, A; Planer-Friedrich, B; Feldmann, J	Biovolatilisation: a poorly studied pathway of the arsenic biogeoc	59	10.1039/c3em00105a
2013	Ahmadjian 1995	8%	Peer-reviewed article	Fungal Biology	Beckett, Richard P ; Zavarzina, Anna G ; Liers, Christi	Oxidoreductases and cellulases in lichens: Possible roles in lichen	32	10.1016/j.funbio.2013.04.007
2013	Larson 1987	8%	Peer-reviewed article	New Zealand Journal of Botany	Dent, JM; Curran, TJ; Rafat, A; Buckley, HL	Microhabitat variation in *Usnea* biomass on mountain beech in N	1	10.1080/0028825X.2013.825633
2013	Larson 1987	8%	Peer-reviewed article	BMC Genomics	Junttila, Sini ; Laiho, Asta ; Gyenesei, Attila ; Rudd, St	Whole transcriptome characterization of the effects of dehydratic	24	10.1186/1471-2164-14-870
2014	Nash 2008	6%	Peer-reviewed article	Botanical Studies	O.W. Purvis	Adaptation and interaction of saxicolous crustose lichens with me	18	10.1186/1999-3110-55-23
2014	Ahmadjian 1995	8%	Peer-reviewed article	Mycobiology	Park, S; Jang, S; Oh, S; Kim, JA; Hur, J	An easy, rapid, and cost-effective method for DNA extraction fror	17	10.5941/MYCO.2014.42.4.311
2014	Ahmadjian 1995 and Haas and Purvis	8%	Peer-reviewed article	Canadian Journal of Microbiolo	Sigurbjornsdottir, MA; Heidmarsson, S; Jonsdottir, A	Novel bacteria associated with Arctic seashore lichens have poter	23	10.1139/cjm-2013-0888
2014	Ahmadjian 1995	10%	Peer-reviewed article	PLoS One	Kumar, J; Dhar, P; Tayade, AB; Gupta, D; Chaurasia,	Antioxidant capacities, phenolic profile, and cytotoxic effects of sa	26	10.1371/journal.pone.0098696
2015	Ahmadjian 1995	8%	Peer-reviewed article	Frontiers in Microbiology	Erlacher, A; Cernava, T; Cardinale, M; Soh, J; Sensen	*Rhizobiales* as functional and endosymbiotic members in the lich	122	10.3389/fmicb.2015.00053
2016	Ahmadjian 1995	8%	Peer-reviewed article	World J Microbiol Biotechnol	Sigurbjornsdottir, MA; Andresson, OS; Vilhelmsson,	Nutrient scavenging activity and antagonistic factors of non-phot	22	10.1007/s11274-016-2019-2
2016	Larson 1987	8%	Peer-reviewed article	Arthropod-Plant Interactions	Goga, Michal; Pöykkö, Heikki; Adlassnig, Wolfram; B	Response of the lichen-eating moth Cleorodes lichenaria larvae to	5	10.1007/s11829-015-9409-5
2017	Larson 1987	8%	Peer-reviewed article	AIMS Geosciences	Schwartzman, DW	Life's Critical Role in the Long-term Carbon Cycle: the Biotic Enhar	10	10.3934/geosci.2017.2.216
2017	Nash 2008	8%	Peer-reviewed article	Biological Reviews	Asplund, J; Wardle, D	How lichens impact on terrestrial community and ecosystem prop	137	10.1111/brv.12305
2017	Larson 1987	8-10%	Peer-reviewed article	Microbiology	Pankratov, T. A ; Kachalkin, A. V ; Korchikov, E. S ; Do	Microbial communities of lichens	16	10.1134/S0026261717030134
2018	no citation	6%	Peer-reviewed article	Czech Polar Reports	Anzhella Valerjevna Sonina* , Vera Ivanovna Andros	Comparative study of structural and ecophysiological features of	1	10.5817/CPR2018-2-15
2018	Ahmadjian 1995 and Nash 2008	8%	Peer-reviewed article	Molecules	Almendras, K; Leiva, D; Caru, M; Orlando, J	Carbon consumption patterns of microbial communities associate	6	10.3390/molecules23112746
2018	Ahmadjian 1995	"a substantial po	Peer-reviewed article	Frontiers in Microbiology	Cernava, T; Vasfiu, Q; Erlacher, A; Aschenbrenner, I/	Adaptations of lichen microbiota functioning under persistent exp	10	10.3389/fmicb.2018.02959
2019	Larson 1987	8%	Peer-reviewed article	Planetary and Space Science	Backhaus, T; Meeben, J; Demets, R; de Vera, JPP; Ot	using Randomly Amplified Polymorphic DNA (RAPD) technique	1	10.1016/j.pss.2019.07.002
2019	Larson 1987	8%	Peer-reviewed article	South African Journal of Botany	Piznak, M; Kolarcik, V; Goga, M; Backor, M	Allelopathic effects of lichen metabolite usnic acid on growth and	2	10.1016/j.sajb.2019.04.011
2019	Larson 1987	8%	Peer-reviewed article	South African Journal of Botany	Piznak, M; Backor, M	Lichens affect boreal forest ecology and plant metabolism	3	10.1016/j.sajb.2019.06.025
2019	Larson 1987	8%	Peer-reviewed article	Journal of Vegetation Science	Nystuen, KO, Sundsdal, K; Opedal, OH; Holien, H; Str	Lichens facilitate seedling recruitment in alpine heath	14	10.1111/jvs.12773
2019	Larson 1987	8%	Peer-reviewed article	Botanica Serbica	Bil'Ova, I; Goga, M; Backor, M	Physiological responses of Xanthoria parietina to longterm coppe	1	10.2298/BOTSERB1902133B
2019	Asplund & Wardle 2017	6-8%	Peer-reviewed article	Biological Reviews	Naranjo-Ortiz, MA; Gabaldon, T	Fungal evolution: major ecological adaptations and evolutionary t	118	10.1111/brv.12510
2020	Larson 1987	7%	Peer-reviewed article	eLife	Fang, H; Labandeira, CC; Ma, Y; Zheng, B; Ren, D; W	Lichen mimesis in mid-Mesozoic lacewings	16	10.7554/eLife.59007
2020	Ahmadjian 1995	8%	Peer-reviewed article	Journal of King Saud University	Rajaram, SK; Ahmad, P; Keerthana, SSS; Cressida, PJ	from lichen associated Streptomyces olivaceus LEP7 against	10	https://www.webofscience.com/wos/woscc/full-record/WOS:000528950800033
2020	Honegger 2006	~10%	Peer-reviewed article	Plant Cell and Environment	Potkay, A; ten Veldhuis, M; Fan, Y; Mattos, CRC; Ana	Water and vapor transport in algal-fungal lichen: Modeling constr	2	10.1111/pce.13690

Peer-reviewed works continued

2020	Larson 1987	~7%	Peer-reviewed article	Geobiology	Nelsen, MP; Lucking, R; Boyce, CK; Lumbsch, HT; Re	No support for the emergence of lichens prior to the evolution of	33	10.1111/gbi.12369
2020	Larson 1987	~7%	Peer-reviewed article	PNAS	Nelsen, MP; Lucking, R; Boyce, CK; Lumbsch, HT; Re	The macroevolutionary dynamics of symbiotic and phenotypic div	31	10.1073/pnas.2001913117
2020	Larson 1987	c. 8%	Peer-reviewed article	Botanical Journal of the Linnea	Xu, M; De Boer, H; Olafsdottir, ES; Omarsdottir, S; H	Phylogenetic diversity of the lichenized algal genus Trebouxia (Tre	15	10.1093/botlinnean/boaa050
2021	no citation	7%	Peer-reviewed article	Diversity (MDPI)	Mei-Xia Yang , Shiva Devkota , Li-Song Wang, and Ch	Ethnolichenology—The Use of Lichens in the Himalayas and South	10	10.3390/d13070330
2021	Ahmadjian 1995	8%	Peer-reviewed article	Microbial Ecology	Jakub Grzesiak & Aleksandra Woltyńska & Marek K.	Metabolic fingerprinting of the Antarctic cyanolichen *Leptogium p*	5	10.1007/s00248-021-01701-2
2021	Ahmadjian 1995	8%	Peer-reviewed article	Microbiome	Wisnu Adi Wicaksono , Peter Kusstatscher , Sabine E	Antimicrobial-specific response from resistance gene carriers stu	7	10.1186/s40168-020-00982-y
2021	Ahmadjian 1995	8%	Peer-reviewed article	Microorganisms (MDPI)	Hyun-Ju Noh, Yerin Park, Soon Gyu Hong, and Yung	Diversity and Physiological Characteristics of Antarctic Lichens-As	14	10.3390/microorganisms9030607
2021	Larson 1987	~7%	Peer-reviewed article	Plants (MDPI)	Francisco Gasulla *, Eva M del Campo, Leonardo M.	Advances in Understanding of Desiccation Tolerance of Lichens a	16	10.3390/plants10040807
2021	Larson 1987	~8%	Peer-reviewed article	Phytochemistry	Masashi A. Ikeda a,* , Hideto Nakamura b , Ken Saw	Long-chain alkenes and alkadienes of eight lichen species collecte	2	10.1016/j.phytochem.2021.112823
2021	Ahmadjian 1995	c. 8%	Peer-reviewed article	The Lichenologist	Beckett, RP; Minibayeva, F; Roach, T	Photoprotection in lichens: adaptations of photobionts to high lig	24	10.1017/S0024282920000535
2021	Larson 1987	c. 8%	Peer-reviewed article	The Lichenologist	Mónika Sinigla , Erzsébet Szurdoki , László Lőkös , Dé	Distribution and habitat preference of protected reindeer lichen s	0	10.1017/S0024282921000165
2022	Ahmadjian 1995	7%	Peer-reviewed article	Environment International	Wisnu Adi Wicaksono, Maria Braun, J˙org Bernhardt,	Trade-off for survival: Microbiome response to chemical exposure	1	10.1016/j.envint.2022.107474
2022	Larson 1987	7%	Peer-reviewed article	Microorganisms (MDPI)	Irene Bruñas Gómez, Monica Casale, Eva Barreno an	Near-Infrared Metabolomic Fingerprinting Study of Lichen Thalli a	1	10.3390/microorganisms10122444
2022	no citation	7%	Peer-reviewed article	Canadian Journal of Forest Res	Marta Alonso-García and Juan Carlos Villarreal Aguil	Bacterial community of reindeer lichens differs between norther	1	10.1139/cjfr-2021-0272
2022	Ahmadjian 1995	8%	Peer-reviewed article	Journal of Ethnopharmacology	Olubukola Tolulope Adenubi, Ibukun Michael Famuy	Lichens: An update on their ethnopharmacological uses and pote	5	10.1016/j.jep.2022.115657
2022	Ahmadjian 1995	8%	Peer-reviewed article	Fungal Biology	Phinney, NH; Asplund, J and Gauslaa, Y	The lichen cushion: A functional perspective of color and size of a	3	10.1016/j.funbio.2022.03.001
2022	Ahmadjian 1995	8%	Peer-reviewed article	Kastamonu University Journal c	Semiha KÖPRÜ, Fatma DOKAN, Zekiye KOCAKAYA, S	Determination of Trace Elements of Some Cladonia Species from	0	10.17475/kastorman.1179052
2022	Larson 1987	6-8%	Peer-reviewed article	Remote Sensing of Environmen	Rasmus Erlandsson, Jarle W. Bjerke, Eirik A. Finne, R	An artificial intelligence approach to remotely assess pale lichen b	2	10.1016/j.rse.2022.113201
2023	Ahmadjian 1995	8%	Peer-reviewed article	FEMS Microbiology Ecology	Aleksandra Wolty´nska, Jan Gawor, Maria A. Olech,	Bacterial communities of Antarctic lichens explored by gDNA and	0	10.1093/femsec/fiad015
2023	Ahmadjian 1995 and Nash 2008	8%	Peer-reviewed article	Ecological Indicators	Carlos Cerrej́on, Osvaldo Valeria, Nicole J. Fenton	Estimating lichen α- and β-diversity using satellite data at differen	0	10.1016/j.ecolind.2023.110173

Books and non-peer-reviewed academic documents

Date published	Citation	Percent coverage reported	Article type	Publication	Authors	Title	Times Cited	DOI or ISBN
2010	cites Ahmadjian 1995	8%	Book chapter	Tools for Identifying Biodiversity: P	Grube, M; Muggia, L	Identifying algal symbionts in lichen symbioses	23	978-88-8303-295-0
1993	cites Larson 1987	8%	Book	Wiley, New York	Ahmadjian, V	The Lichen Symbiosis	1202	471578851
2008	cites Larson 1987 and Ahmadjian 1995	8-10%	Textbook	Cambridge Press	edited by Nash, authors chapters worth.	Lichen Biology	unknown	521692164
1995	cites Larson, no date	8%	Letter	Biosciences	Ahmadjian, V	Lichens are more important than you think	45	10.1093/bioscience/45.3.124
2001	no citation	8%	Textbook	Yale University Press	Irwin M Brodo, Stephen Sharnoff, Sylvia [	Lichens of North America	unknown	9.7803E+12
2008	no citation	6%	Textbook chapter	Encyclopedia of Ecology	Gadd, G.M.	Fungi and their role in the biosphere	46	10.1016/B978-008045405-4.00734-5
2012	no citation	6%	Textbook chapter	Ecology of Industrial Pollution	O.W. Purvis	Lichens and industrial pollution	13	1139486160
2012	no citation	6%	Conference abstrac	Geophysical Research Abstracts	O.W. Purvis, P. Convey, M.J. Flowerdew,	Lichens and weathering: importance for soil for	2	2012EGUGA..14.2802P
2018	no citation	6%	Doctoral thesis	Frankfurt am Main	Bastian Greshake Tzovaras	Characterizing the hologenome of Lasallia pustu	unknown	https://publikationen.ub.uni-frankfurt.de/frontdoor/index/index/docId/45701
2006	no clear citation	6%	Textbook section	Fungi in Biogeochemical Cycles	edited by Gadd; Haas, JR; Purvis, OW	Lichen biogeochemistry	136	521845793
2006	no clear citation	c. 10%	Textbook section	Fungi in the Environment	edited by Gadd; Honegger, R	Water relations in lichens	61	1139462105
2020	no citation	book	Book	Random House	Merlin Sheldrake	Entangled Life	unknown	pg. 74
Mar-21	no citation?	book	Book	Renaud-Bray	Vincent Zonca	Lichens: pour une resistance minimale	unknown	https://www.renaud-bray.com/Livres_Produit.aspx?id=3327861&def=Lichens+%3A+pour+une+r%C3%A9sistance+minimale%2CZONCA%2C+VINCENT%2C9782746521988

Larson 1987 citations not for 8% statistic

**Authors**	**Title**	**Journal info**	**Year Only**	Times Cited	**Notes**
Egan, R.S.	RECENT LITERATURE ON LICHENS-131	Bryologist, 1987, Vol. 90 (4), p. 456-465	1987	1	listing only - summary does not include dominance statistic
D. S. Coxson ; D. D. McIntyre ; H. J. Vogel	PULSE RELEASE OF SUGARS AND POLYOLS FROM CANOPY BR	Biotropica, 1992-06-01, Vol.24 (2), p.121-133	1992	47	
Souza-Egipsy, Virginia ; Valladares, Fernando	Water distribution in foliose lichen species: Interactions betw	Annals of botany, 2000-09, Vol.86 (3), p.595-601	1993	20	
Honegger, Rosmarie	Developmental biology of lichens	The New phytologist, 1993-12, Vol.125 (4), p.659-677	1993	165	
Esteban Manrique ; Luis Balaguer ; Jeremy Ba	PHOTOINHIBITION STUDIES IN LICHENS USING CHLOROPHYLI	The Bryologist, 1993-10-01, Vol.96 (3), p.443-449	1993	35	
Fos, Simón ; Deltoro, Vicente I ; Calatayud, Án	Changes in water economy in relation to anatomical and mor	The Lichenologist (London), 1999-07, Vol.31 (4), p.375-387	1999	14	
Gomez-Pujol, Lluis; Stephenson, Wayne J.; Fo	Two-hourly surface change on supra-tidal rock (Marengo, Vic	Earth surface processes and landforms, 2007-01, Vol.32 (1), p.1-12	2007	36	
BACKOR, M ; FAHSELT, D	Lichen photobionts and metal toxicity	Symbiosis (Philadelphia, Pa.), 2008, Vol.46 (1), p.1-10	2008	32	
Hassan, Ammar Ali ; Brustad, Magritt ; Sandar	Concentrations and geographical variations of selected toxic	International journal of environmental research and public health, 2012-05	2012	8	Spelled Larson as "Larsen"
Ali Hassan, Ammar ; Rylander, Charlotta ; Bru	Level of selected toxic elements in meat, liver, tallow and	International journal of circumpolar health, 2012-01-31, Vol.71 (1), p.1-7	2012	16	Spelled Larson as "Larsen"
Kachalkin, A V ; Glushakova, A M ; Pankratov,	Yeast population of the Kindo Peninsula lichens	Microbiology (New York), 2017-11, Vol.86 (6), p.786-792	2017	2	
LONGINOTTI, Sara ; SOLHAUG, Knut Asbjørn ;	Hydration traits in cephalolichen members of the epiphytic old	The Lichenologist (London), 2017-09, Vol.49 (5), p.493-506	2017	17	
Gauslaa, Yngvar ; Solhaug, Knut Asbjørn ; Lon	Functional traits prolonging photosynthetically active periods	Environmental and experimental botany, 2017-09, Vol.141, p.83-91	2017	24	
Bianchi, Elisabetta ; Benesperi, Renato ; Colzi,	The multi-purpose role of hairiness in the lichens of coastal er	Plant physiology and biochemistry, 2019-08, Vol.141, p.398-406	2019	9	
Yuan, Runjie ; Kennedy, David M ; Stephensor	Experimental investigations into the influence of biofilms and	Earth surface processes and landforms, 2019-06-15, Vol.44 (7), p.1377-138	2019	5	
Maria Grimm, Martin Grube, Ulf Schiefelbein,	The Lichens' Microbiota, Still a Mystery?	Front. Microbiol., 30 March 2021 | https://doi.org/10.3389/fmicb.2021.62	2021	7	
Zi-bo Li , Lianwen Liu, Xiancai Lu, Liang Zhao,	Mineral foraging and etching by the fungus Talaromyces flavu	Chemical Geology 586 (2021) 120592	2021	3	
Matthew P. Nelsen, Steven D. Leavitt, Kathlee	Macroecological diversification and convergence in a clade of	FEMS Microbiology Ecology, 2021, Vol.97(6), p. fiab072	2021	11	
Coxson, DW	Impedance measurement of thallus moisture content in liche	Lichenologist, 1991, Vol.23(1), p. 77-84	1991	30	

Ahmadjian 1995 citations not for 8% statistic

Authors	Title	DOI or Journal info	Year Only	Times Cited	Notes
Toma´sˇ Rˇzanka; Irene A. Guschina	Brominated Depsidones from Acarospora g	J. Nat. Prod. 1999, 62, 12, 1	1999	23	
Hammer, S	Lateral growth patterns in the Cladoniacea	American journal of botany	2001	6	
Inoue, T; Kudoh, S; Kanda, H	Effects of substrate differences on water a	Polar science. , 2014, Vol.8(	2014	4	
Li, H; Wei, JC	Functional analysis of thioredoxin from the	Scientific reports. , 2016, V	2016	8	
Inoue, T; Kudoh, S; Kanda, H	Factors affecting water availability for high	Polar biology , 2017, Vol.40	2017	1	
Cernava, T; Aschenbrenner, IA; Berg, G	Plasticity of a holobiont: desiccation induce	The ISME journal. , 2019, V	2019	25	
Allen, JL; McMullin, RT; Lendemer, JC	Lichen conservation in North America: a re	Biodiversity and conservati	2019	27	
Exposito, JR; Coello, AJ; Catala, M	Endogenous NO Is Involved in Dissimilar Re	Microbial ecology. , 2020, V	2020	4	
Sanders, William B.; Masumoto, Hiroshi	Lichen algae: the photosynthetic partners i	The Lichenologist. , 2021, V	2021	22	
Julian Klein; Matthew Low; Go¨ran Thor; Jo¨rgen Sjo	Tree species identity and composition shap	PLoS ONE	2021	7	
S. Elizabeth Arnold	Mycology: Metagenomes illuminate evolut	10.1016/j.cub.2022.10.041	2023	0	
Tran Van Cuong, Se-Young Cho, Joseph Kwon, and Du	Elucidation of the Inhibitory Mechanisms o	J. Microbiol. Biotechnol., vo	2019	1	

Inaccessible articles (at the time of this publication)

Citation	Authors	Title	Journal info	Times Cited
cites Larson 1987	Arumugam D. Gandhi, Sivaji Sathiyaraj, Guna	Lichens in Genus Parmelia: An Overview and their A	21(13), DOI :	1
cites Larson 1987	Valladares, F ; Sancho, L. G ; Ascaso, Carmen	Water storage in the Lichen family Umbilicariaceae	Botanica Acta, 1998-04, Vol.111 (2), p.99-107	28
cites Larson 1987	Vicente, C	THE CONCEPT OF ENDOHABITAT IN PHYCOBIONT-CO	Endocytobiosis and Cell Research (1990), 7(1-2): 61-7	7

Popular or other media

Date published	Citation	Media type	Publication	Authors	Title	Link or page #
unknown	no citation	commerce web	Keep It Handsome	unknown	What is lichen? Lichen in KIH products	https://www.keepithandsome.ca/pages/lichen
05-Jan-21	cites Asplund & Wardle 2017	Encyclopedia	Wikipedia	unknown	Lichen (wiki entry)	https://en.wikipedia.org/wiki/Lichen
year 2022	no citation (but from a recent paper from the lab	lab update	Villarreal Lab	unknown	Microbiome of reindeer lichens in Eastern North	https://villarreal-lab.ibis.ulaval.ca/2022/01/23/microbiome-of-reindeer-lichens-in-eastern-north-america/
unknown	no citation	lesson plan	Wildsight	"Patty"	The world of lichen	https://wildsight.ca/education-resources/the-world-of-lichen/
16-Jan-20	cites Gadd 2010, which cites Haas & Purvis 2006	popular article	Forbes	Linh Anh Cat	Giant lichens can grow to the size of dinner plate	https://www.forbes.com/sites/linhanhcat/2020/01/16/giant-lichens-dinner-plate-sized/?sh=7ac85b1154c4
05-Feb-20	no citation	popular article	UWM Field Statio	"The BugLady"	You'll be lichen this article	https://uwm.edu/field-station/youll-be-lichen-this-article/
22-Jul-16	no citation	popular article	Science	Elizabeth Pennisi	A lichen ménage à trois	https://science.sciencemag.org/content/353/6297/337.summary#:~:text=Summary,Earth's%20surface%2C%20by%20one%20estimate.
13-Feb-22	no citation	popular article	Observer	Emma Roth	Lichens offer lesson in cooperation	https://www.observertoday.com/news/page-one/2022/02/lichens-offer-lesson-in-cooperation/
12-May-20	no citation	popular article	Treehugger	Jaymi Heimbuch	The Unexpectedly Weird and Beautiful World of	https://www.treehugger.com/the-unexpectedly-weird-and-beautiful-world-of-lichens-4863470
unknown	no citation	popular article	Goodness Exchan	Liesl Ulrich-Verde	Little lichen wonders of the world	https://goodness-exchange.com/what-is-lichen/
24-Jul-201	no citation	popular article	Science (The Wire	Nandita Jayaraj	Has a missing piece of the lichen puzzle been fou	https://science.thewire.in/science/lichen-photobiont-yeast-biochemistry/
22-Jan-21	no citation	popular article	BBC Two	unknown	Liking lichens: What to watch this winter	https://www.bbc.co.uk/blogs/natureuk/entries/a68831ab-3d51-4dee-ab81-e5d976033e44?fbclid=IwAR2E6WplfZtR6i9YLro6ANKcb9MXP8unrdAcbLqcpIZIK6cR4qb69rpjRuM
2022-02-15	no citation	popular article	Scientific America	Jack Tamisiea	Lichens could need more than a million years to	https://www.scientificamerican.com/article/lichens-could-need-more-than-a-million-years-to-adapt-to-climate-change/
20-Nov-19	no citation (but likely from Nelsen et al. 2020)	popular article	Science	Eva Frederick	Hardy lichens don't actually predate plants	https://www.science.org/content/article/hardy-lichens-don-t-actually-predate-plants
19-Nov-19	no citation (but likely from Nelsen et al. 2020)	popular article	New York Times	JoAnna Klein	In the Race to Live on Land, Lichens Didn't Beat	https://www.nytimes.com/2019/11/19/science/lichens-plants-evolution.html
15-Feb-22	no citation (but likely from Nelsen et al. 2022)	popular article	Cosmos	Amalyah Hart	A poor climate for liking lichens	https://cosmosmagazine.com/science/biology/lichens-losing-race-climate-change/
15-Feb-22	no citation (but likely from Nelsen et al. 2022)	popular article	CNN	Ashley Strickland	Earth is heating up too quickly for these tiny org	https://www.cnn.com/2022/02/15/world/lichen-algae-climate-change-scn/index.html
16-Feb-22	no citation (but likely from Nelsen et al. 2022)	popular article	ZME Science	Fermin Koop	lichens are having a hard time catching up to cli	https://www.zmescience.com/science/lichens-difficult-time-catch-up-climate-change-15022022/
15-Feb-22	no citation (but likely from Nelsen et al. 2022)	popular article	EurekAlert! AAAS	unknown	Lichens are in danger of losing the evolutionary	https://www.eurekalert.org/news-releases/942949
Jan-22	no citation?	radio	BBC 4	unknown	Fungi: The New Frontier	https://www.bbc.co.uk/programmes/m00132xn
09-Jan-18	no citation	radio (w/ accon	WXPR	Scott Bowe	Lichens - What's not to like?	https://www.wxpr.org/natural-resources/2018-01-09/lichens-whats-not-to-like
year 2021	no citation	talk advert	Alabama State Pa	unknown	Desota State Park: A world in miniature: lichens	https://www.alapark.com/DSP-CT-Lichens
25-Jan-18	no citation	Video	National Geograp	Tim Wheeler	What's in a lichen? How Scientists Got it Wrong	https://www.youtube.com/watch?v=Fkw_VF5zDT0
17-Jan-21	no citation	Video	The Naturalist	unknown	Lichens: Marvelous Composite Organisms	1:05 in - https://www.youtube.com/watch?v=IPqgnPl6Kfg&feature=youtu.be&ab_channel=TheNaturalist
Jun-20	no citation	webinar advert	Bay Nature	Jesse Miller	Webinar: Maintaining California Lichen Diversity	https://baynature.org/event/webinar-maintaining-california-lichen-diversity-in-an-era-of-global-change/
unknown	citation, but ref list not included	website	Fungi from India	unknown	Lichens	http://www.fungifromindia.com/fungiFromIndia/databases/ILD/
18-Aug-11	cites Ahmadjian 1995	website	Australian Nation	Heino Lepp	Lichen - Ecology - Habitats	https://www.anbg.gov.au/lichen/ecology-habitats.html
21-Sep-20	no citation	website	London Wildlife T	"Keeping it wild" t	Learning about lichen	https://www.wildlondon.org.uk/blog/keeping-it-wild-project/learning-about-lichen-keeping-it-wild-trainee-robert
20-Dec-21	no citation	website	Fingle Woods	Jane Halliday	Alive with lichen	https://finglewoods.org.uk/2021/12/20/alive-with-lichen/
year 2019	no citation	website	Game & Wildlife (	Peter Thompson	Lichen	https://www.gwct.org.uk/wildlife/species-of-the-month/2019/lichen/
06-Apr-21	no citation	website	University of Mar	Sara Tangren	Lichens	https://extension.umd.edu/resource/lichens
17-May-21	no citation	website	Schlitz Audubon N	Tess Stahler	Survival Strategies of Lichen	https://www.schlitzaudubon.org/2021/05/17/survival-strategies-of-lichen/
unknown	no citation	website	Duchy of Cornwal	unknown	Lichens	https://www.duchyofcornwallnursery.co.uk/journal/lichens
02-Jul-20	no citation	website	Georgian Bay Bios	unknown	Are you likin' the lichen?	https://www.gbbr.ca/are-you-likin-the-lichen/#:~:text=There%20are%20over%2020%2C000%20different,way%20to%20the%20frozen%20tundra.
unknown	no citation	website	LAM Tree Service	unknown	Lichen on your trees: good or bad?	https://www.lamtree.com/lichen-on-your-trees-good-or-bad/
2015	no citation	website	Sky Meadows	unknown	Lichens	https://www.skymeadows.info/lichens
unknown	no citation	website	de rubus plantaru	unknown	Myths and truths about lichens	https://drp.bio/en/myths-and-truths-about-lichens/
